# Suitability of *Hermetia illucens* larvae meal and fat in broiler diets: effects on animal performance, apparent ileal digestibility, gut histology, and microbial metabolites

**DOI:** 10.1186/s40104-022-00701-7

**Published:** 2022-05-09

**Authors:** Kristina Hartinger, Katharina Fröschl, Marco Antonio Ebbing, Barbara Bruschek-Pfleger, Karl Schedle, Christiane Schwarz, Martin Gierus

**Affiliations:** Department of Agrobiotechnology, Institute of Animal Nutrition, Livestock Products, and Nutrition Physiology, University of Natural Resources and Life Sciences, Vienna, Austria

**Keywords:** Broiler chicken, *Hermetia illucens*, Larvae fat, Larvae meal, Lauric acid, Microbial metabolites, Performance

## Abstract

**Background:**

The possibility of partially replacing soybean meal (SBM) with *Hermetia illucens* (HI) defatted larvae meal in broiler nutrition has frequently been suggested. For sustainability reasons, however, the larvae fat produced during defatting should also be used and could be particularly beneficial regarding gut health due to its fatty acid composition. To evaluate the suitability of HI larvae as protein and fat source, a 2 × 3 factorial arrangement with two types of protein, i.e. SBM (S) or SBM and 15% of its crude protein replaced by HI larvae meal (L), and three levels of fat sources, namely 0 (0 L), 50% (50 L) or 100% HI larvae fat (100 L) at the expense of soybean oil was applied.

**Results:**

In the starter phase, an interaction showed higher body weight (BW), average daily gain (ADG) and improved feed conversion ratio (FCR) if 50% or 100% HI larvae fat was fed with HI larvae meal. Moreover, BW, ADG and FCR improved when feeding HI larvae meal as protein source. Additionally, we observed an increased average daily feed intake in the grower, finisher, and overall phase in the L groups and an improved FCR in 0 L compared to 50 L groups during the overall period. Regarding apparent ileal digestibility, HI larvae meal feeding increased dry matter, organic matter, and fat digestibility. Feeding HI larvae meal as protein source decreased the concentrations of agmatine, spermidine, spermine and ammonia in the caecal digesta, whereas fat source affected agmatine with higher concentrations in 50 L compared to 0 L in the colonic digesta. In contrast, caecal ethanolamine concentrations increased in HI larvae meal groups compared to SBM. Caecal butyric acid concentrations decreased with HI larvae meal feeding. An interaction was found for the jejunal villus area, being higher in L + 100 L compared to S + 100 L. Furthermore, L groups had greater villus width.

**Conclusions:**

A partial replacement of SBM with HI larvae meal and soybean oil with HI larvae fat in broiler diets without impairing animal performance or gut health seems possible. Feeding HI larvae meal affected broiler performance positively in the starter phase and improved apparent ileal digestibility.

## Introduction

To cover the high demand for protein-rich feeds in European poultry production, soybean meal (SBM) imports, in particular from South America, currently represent the largest source [1, 2]. Due to the strong import dependency on this protein source, keen interest exists on investigations of insects as alternative protein source. Insects constitute a natural part of poultry diets in wildlife [3, 4] and are seen as a possible alternative valuable feedstuff in animal nutrition [5–9]. The larvae of the black soldier fly (*Hermetia illucens*, HI) are rich in crude protein (CP) and fat, with levels of about 42% and 15–37% in dry matter (DM), respectively [10, 11]. Moreover, CP content can be further increased by defatting, in order to generate a protein meal [10]. Additionally, separation of protein and fat may enable a more balanced and precise diet formulation. However, usage of the remaining larvae fat should be considered with respect to sustainability reasons and because of potential positive nutritionally and antimicrobial effects of the fatty acids [12]. *Hermetia illucens* larvae fat is characterised by saturated medium-chain fatty acids (MCFA) (C6:0-C12:0), of which lauric acid (C12:0) accounts for the largest proportion of more than 50% of total fatty acids [13–15]. Medium-chain-fatty acids are regarded as readily digestible, since they do not require bile acid-mediated micelle formation to emulsify in the aqueous phase of digesta, but are absorbed by simple passive diffusion and thus an immediate energy source e.g. for enterocytes [12, 16, 17]. Consequently, positive effects on nutrient digestibility and intestinal morphology may result, which need to be investigated. Besides, blending animal fats with vegetable oils may have synergistic effects, mainly arising from balanced ratios of saturated and unsaturated fatty acids [18]. Furthermore, MCFA, especially lauric acid, are ascribed a potentially favourable antimicrobial effect by disrupting the bacterial cell electron transport chain and consequently impairing the energy supply [12, 19, 20]. By influencing the microbial composition, effects of MCFA on the microbial fermentation profile can also result [21] and investigating microbial metabolites, such as biogenic amines and ammonia, as well as short-chain fatty acids (SCFA) that derive from protein or carbohydrate fermentation, could provide knowledge about the effects of HI larvae fat.

In accordance with others, we have previously shown that HI larvae meal, used in small quantities (5% in diet fresh matter), is suitable to reduce SBM in broiler feeds without detrimental effects on performance, apparent ileal amino acid digestibility and histology [22–25]. Regarding the use of insect fats in broiler feeding, complete replacement of soybean oil with HI larvae fat seemed possible without adverse impact on growth performance [15, 26–28]. Although more and more studies are being conducted on feeding both HI larvae meal and fat, to the best of our knowledge, there have been no studies on the influence of feeding both components simultaneously but in separated form. Therefore, the present study aimed to document the performance of broilers during the 35-d growing period when fed HI larvae meal and fat in different ratios and assess the impact of HI meal and fat use on nutrient digestibility as well as on gut health variables, i.e. intestinal morphology and microbial metabolites in the caecal and colonic digesta. We hypothesised that the partial replacement of SBM and total replacement of soybean oil with HI larvae meal and fat, respectively, is possible without impairing digestibility and consequently animal performance characteristics. As result of the above-mentioned antimicrobial effects, potentially favourable shifts in the production of microbial metabolites, e.g. biogenic amines and SCFA, may occur. This study is first to describe the effects of simultaneously feeding HI larvae meal and fat on broiler performance, digestibility, and gut health apart from studies feeding whole larvae. It should therefore provide new knowledge on the targeted applicability of HI larvae components as poultry feed.

## Materials and methods

### Birds, housing, and diets

The present trial has been carried out under compliance with the 1^st^ regulation of keeping of animals (BGBl. II Nr. 485/2004) and was approved by the Ethics Committee of the University of Natural Resources and Life Sciences, Vienna with the reference number 2021/006. It took place at a poultry research station rented by the University of Natural Resources and Life Sciences, Vienna, Austria. All environmental conditions, such as ambient temperature, lighting scheme or humidity, were adjusted to meet standard breeding practices ([29] Aviagen: Ross broiler management handbook) and described previously in Hartinger et al. [25]. In total, 432, one-day-old Ross 308 male broiler chickens were randomly assigned to one of six dietary treatments and allotted to 36 pens with 12 animals per pen, resulting in six replicates per treatment. Each pen was equipped with a feeder, an automatic drinker and wood shavings as litter material. Birds had an initial BW of 42.5 g (±0.63 g) and were purchased from a commercial local hatchery (Geflügelhof Laßnitzhöhe GmbH, Laßnitzhöhe, Austria). All diets were formulated according to the breeder’s nutritional specifications [30] within a three phases feeding program: starter diet was fed from d 1 to d 14, grower diet from d 15 to d 28 and finisher diet from d 29 to d 36. The HI larvae meal and fat were obtained from a commercial company (Ecofly GmbH, Antiesenhofen, Austria) and their nutrient composition is shown in Table 1. As data concerning larvae meal amino acid digestibility are scarce and inconsistent, diets were calculated based on total amino acids (Ross 308 Broiler Nutrition Specifications (2019)) [30]. The experimental design was a 2 × 3 factorial arrangement with two types of protein, i.e. either SBM solely (S) or SBM and 15% of its CP replaced by HI larvae meal (L), as well as three levels of fat sources, namely 0 HI larvae fat (0 L), 50% HI larvae fat (50 L) or 100% HI larvae fat (100 L) at the expense of soybean oil (Table 2). Therefore, the six treatments were: S + 0 L, S + 50 L, S + 100 L, L + 0 L, L + 50 L, L + 100 L. During all phases, diets were formulated to be isoenergetic and isonitrogenous (Table 3) and were provided ad libitum. To determine the ileal digestibility, titanium dioxide was included (3 g/kg fresh matter) in the finisher feeds. The starter diet was fed in crumbled form (granulation gap 1.7 mm), whereas grower (2.3 mm) and finisher (2.8 mm) diets were offered in pelleted form.

**Table 1:** Proximate analysis (% of dry matter) and fatty acid composition of defatted *Hermetia illucens* larvae meal and *Hermetia illucens* larvae fat

Items	Larvae meal	Larvae fat
Dry matter, %	95.2	–
Crude protein, %	67.6	–
Acid-detergent fibre (ADF), %	17.9	–
ADF-linked protein, %	10.2	
Ash, %	8.79	–
Ether extract following acid hydrolysis, %	9.52	–
Lysine, %	3.42	–
Methionine, %	1.14	–
Cysteine, %	9.8	–
Threonine, %	2.39	–
Phosphorus, g/kg	10.1	–
Calcium, g/kg	9.54	–
Sodium, g/kg	1.19	–
Fatty acid composition, % of total FAME		
C 10:0 (Capric acid)	0.99	1.25
C 11:0 (Undecanoic acid)	–	0.12
C 12:0 (Lauric acid)	43.65	54.42
C 14:0 (Myristic acid)	8.71	10.13
C 16:0 (Palmitic acid)	15.06	12.22
C 18:0 (Stearic acid)	2.87	1.73
Total SFA	72.13	80.90
C 16:1 (Palmitoleic acid)	2.90	2.36
C 18:1 n-9 t (Elaidic acid)	–	0.18
C18:1 n-9 (Oleic acid)	13.14	7.92
Total MUFA	16.04	10.78
C 18:2 n-6c (Linoleic acid)	10.97	7.67
C 18:3 n-3 (α-Linolenic acid)	0.86	0.65
Total PUFA	11.83	8.33
UFA/SFA	0.39	0.24

*FAME* Fatty acid methyl ester, *SFA* Saturated fatty acids, *MUFA* Monounsaturated fatty acids, *PUFA* Polyunsaturated fatty acids, *UFA* Unsaturated fatty acids

**Table 2:** Composition of experimental diets

Ingredients, g/kg as fed	Starter period (d 1–14)						Grower period (d 15–28)						Finisher period (d 29–35)					
	S + 0 L	S + 50 L	S + 100 L	L + 0 L	L + 50 L	L + 100 L	S + 0 L	S + 50 L	S + 100 L	L + 0 L	L + 50 L	L + 100 L	S + 0 L	S + 50 L	S + 100 L	L + 0 L	L + 50 L	L + 100 L
Corn	559	559	559	564	564	564	583	583	583	600	600	600	595	595	595	603	603	603
Soybean meal(48% CP)	385	385	385	332	332	332	350	350	350	300	300	300	318.5	318.5	318.5	270	270	270
HI larvae meal	–	–	–	52.0	52.0	52.0	–	–	–	46.4	46.4	46.4	–	–	–	40.0	40.0	40.0
Soybean oil	15.0	7.50	–	12.8	6.40	–	31.4	15.7	–	20.0	10.0	–	51.0	25.5	–	50.0	25.0	–
HI larvae fat	–	7.50	15.0	–	6.40	12.8	–	15.7	31.4	–	10.0	20.0	–	25.5	51.0	–	25.0	50.0
Dicalcium- phosphate	15.5	15.5	15.5	16.0	16.0	16.0	13.0	13.0	13.0	13.0	13.0	13.0	12.0	12.0	12.0	13.0	13.0	13.0
Feed limestone	12.0	12.0	12.0	9.0	9.0	9.0	10.0	10.0	10.0	10.0	10.0	10.0	10.0	10.0	10.0	10.0	10.0	10.0
Mineral premix^1^	0.70	0.70	0.70	0.70	0.70	0.70	0.70	0.70	0.70	0.70	0.70	0.70	0.70	0.70	0.70	0.70	0.70	0.70
Vitamin premix^2^	0.36	0.36	0.36	0.36	0.36	0.36	0.36	0.36	0.36	0.36	0.36	0.36	0.36	0.36	0.36	0.36	0.36	0.36
Salt	4.00	4.00	4.00	4.00	4.00	4.00	4.00	4.00	4.00	4.00	4.00	4.00	4.00	4.00	4.00	4.00	4.00	4.00
L-methionine (99%)	4.00	4.00	4.00	4.00	4.00	4.00	3.50	3.50	3.50	2.40	2.40	2.40	3.00	3.00	3.00	3.20	3.20	3.20
Lysine^3^	2.60	2.60	2.60	2.30	2.30	2.30	1.90	1.90	1.90	1.70	1.70	1.70	1.40	1.40	1.40	1.50	1.50	1.50
L-threonine	1.00	1.00	1.00	1.00	1.00	1.00	0.80	0.80	0.80	0.80	0.80	0.80	0.50	0.50	0.50	0.30	0.30	0.30
L-valine	0.30	0.30	0.30	0.04	0.04	0.04	0.30	0.30	0.30	–	–	–	–	–	–	–	–	–
Arginine	–	–	–	0.50	0.50	0.50	–	–	–	–	–	–	–	–	–	–	–	–
Choline chloride (60%)	0.50	0.50	0.50	0.50	0.50	0.50	0.50	0.50	0.50	0.50	0.50	0.50	0.50	0.50	0.50	0.50	0.50	0.50
Sacox^4^	0.50	0.50	0.50	0.50	0.50	0.50	0.50	0.50	0.50	0.50	0.50	0.50	–	–	–	–	–	–
Optiphos^5^	0.10	0.10	0.10	0.10	0.10	0.10	0.10	0.10	0.10	0.10	0.10	0.10	0.10	0.10	0.10	0.10	0.10	0.10
Titanium dioxide	–	–	–	–	–	–	–	–	–	–	–	–	3.00	3.00	3.00	3.00	3.00	3.00

S + 0 L, SBM + 0HI larvae fat; S + 50 L, SBM + 50% HI larvae fat; S + 100 L, SBM+ 100% HI larvae fat; L + 0 L, HI larvae meal+ 0HI larvae fat; L + 50 L, HI larvae meal+ 50% HI larvae fat; L + 100 L, HI larvae meal+ 100% HI larvae fat ^1^Composition in mg per kg mineral premix: 35.71 Fe, 17.14 Cu, 92.86 Zn, 128.57 Mn, 2.14 I, 571 Se; ^2^Composition in mg per kg vitamin premix: 27,777,778 IE vitamin A, 13,888,889 IE vitamin D_3_, 133,333 vitamin E; 11,111 vitamin K (menadione), 5556 vitamin B_1_, 16,667 vitamin B_2_, 11,111 vitamin B_6_, 55,556 μg/kg vitamin B_12_, 417 biotin, 2778 folic acid, 125,000 niacin, 34,722 pantothenic acid; ^3^HCl-lysine 78% Lysine; ^4^ Salinomycin-Sodium: 114–132 g/kg, silicium dioxide: 10–100 g/kg, calcium carbonate: 500–700 g/kg (Huvepharma); ^5^6-phytase derived from *E. coli* (Huvepharma);

**Table 3:** Analysed nutrient and fatty acid composition of experimental diets

Items	Starter period (d 1–14)						Grower period (d 15–28)						Finisher period (d 29–35)					
	S + 0 L	S + 50 L	S + 100 L	L + 0 L	L + 50 L	L + 100 L	S + 0 L	S + 50 L	S + 100 L	L + 0 L	L + 50 L	L + 100 L	S + 0 L	S + 50 L	S + 100 L	L + 0 L	L + 50 L	L + 100 L
Analysed nutrient composition, % DM																		
Dry matter, % FM	90.0	89.9	89.7	90.2	89.8	90.2	89.3	89.4	89.0	89.5	89.4	89.6	89.4	89.3	89.0	89.0	89.6	89.7
Crude protein	26.9	26.7	26.5	28.1	27.9	27.3	26.1	25.3	25.4	25.6	25.7	25.2	22.9	22.8	23.8	23.1	23.0	24.7
Ether extract^1^	5.9	5.7	5.9	5.8	5.8	5.9	8.1	8.0	7.6	6.7	6.9	6.8	9.7	9.4	9.2	9.6	9.1	9.6
Ash	7.0	6.7	7.1	6.9	6.6	6.8	6.3	6.5	6.5	6.3	6.3	6.2	6.3	5.9	6.0	6.0	6.0	6.4
Sugar	4.9	4.9	5.0	4.2	4.2	4.3	4.7	4.6	4.6	3.9	4.1	4.0	4.2	4.2	4.3	3.1	3.7	3.8
Starch	44.0	45.6	44.1	45.3	45.4	43.9	44.0	44.8	43.6	46.3	46.5	46.0	45.7	46.0	46.6	46.8	47.3	45.8
Calcium	1.16	0.92	0.98	0.85	0.85	0.92	0.79	0.84	0.91	0.91	0.86	0.85	0.86	0.76	0.81	0.80	0.78	0.88
Phosphorous	0.72	0.71	0.73	0.76	0.77	0.76	0.63	0.67	0.69	0.68	0.68	0.66	0.55	0.61	0.63	0.65	0.61	0.65
Sodium	0.23	0.23	0.20	0.20	0.18	0.20	0.20	0.20	0.18	0.19	0.18	0.16	0.19	0.17	0.17	0.17	0.16	0.19
Lysine	1.68	1.70	1.63	1.62	1.77	1.70	1.65	1.57	1.61	1.50	1.51	1.47	1.26	1.24	1.29	1.32	1.17	1.32
Methionine	0.74	0.74	0.77	0.75	0.80	0.81	0.66	0.66	0.60	0.56	0.52	0.49	0.56	0.56	0.59	0.65	0.55	0.57
AME_N_, MJ/kg^2^	12.8	12.9	12.7	13.0	12.9	12.8	13.2	13.2	12.8	13.0	13.1	12.9	13.5	13.4	13.5	13.6	13.5	13.7
Fatty acid composition, % of total FAME																		
C12:0 (Lauric acid)	0.0	7.3	14.9	3.9	10.4	15.5	0.9	12.1	23.4	3.6	11.6	19.3	0.9	14.6	29.6	2.5	15.8	30.7
C14:0 (Myristic acid)	3.70	1.42	2.82	0.84	2.04	3.06	2.16	2.39	4.41	0.71	2.30	3.74	1.53	2.76	5.57	0.59	3.02	5.73
C16:0 (Palmitic acid)	12.7	13.6	13.7	13.6	13.9	14.0	12.5	13.3	13.6	13.3	13.6	13.8	12.2	12.9	13.4	12.6	13.0	13.3
C18:0 (Stearic acid)	2.85	2.64	2.53	2.97	2.84	2.57	3.10	2.65	2.38	3.00	2.70	2.39	3.17	2.79	2.15	3.22	2.74	2.20
Total SFA	19.2	25.9	34.0	21.3	29.2	35.1	19.0	30.4	44.3	20.6	30.2	39.2	18.6	33.6	51.1	19.6	35.1	52.9
C16:1	1.31	2.67	2.21	1.71	1.62	1.48	1.39	1.90	1.58	1.72	1.51	1.06	1.02	1.22	1.96	0.90	1.25	1.90
C18:1 n-9 t (Elaidic acid)	0.0	0.0	0.0	0.0	0.0	0.0	0.0	0.0	0.0	0.0	0.0	0.0	0.0	0.0	0.0	0.0	0.0	0.0
C18:1 n-9 (Oleic acid)	22.1	20.4	18.0	21.9	20.2	18.9	22.6	19.7	16.4	22.6	20.2	18.3	23.1	19.5	14.9	23.1	19.3	14.8
Total MUFA	23.4	23.3	20.7	24.0	21.9	21.0	24.0	21.6	18.3	24.4	21.7	19.4	24.2	20.7	16.9	24.2	26.7	16.8
C18:2 n-6 (Linoleic acid)	52.5	47.3	41.4	50.4	45.1	40.4	52.1	44.0	34.4	50.7	44.3	38.3	52.2	42.0	29.1	51.4	40.7	28.2
C18:3 n-3 (α-Linolenic acid)	3.94	3.38	2.91	3.62	3.05	2.59	4.33	3.31	2.33	3.74	3.07	2.41	4.37	3.30	1.95	4.21	3.13	1.74
Total PUFA	57.4	51.6	45.3	54.8	49.0	43.9	57.0	48.0	37.5	55.1	48.1	41.4	57.2	45.7	31.6	56.2	44.3	30.4
UFA/SFA	4.2	3.0	1.9	3.7	2.4	1.9	4.3	2.3	1.3	3.9	2.3	1.5	4.4	2.0	0.9	4.1	1.8	0.9

S + 0 L, SBM + 0 HI larvae fat; S + 50 L, SBM + 50% HI larvae fat; S + 100 L, SBM+ 100% HI larvae fat; L + 0 L, HI larvae meal+ 0 HI larvae fat; L + 50 L, HI larvae meal+ 50% HI larvae fat; L + 100 L, HI larvae meal+ 100% HI larvae fat FAME, fatty acid methyl esters; *SFA* Saturated fatty acids, *MUFA* Monounsaturated fatty acids, *PUFA* Polyunsaturated fatty acids, *UFA* Unsaturated fatty acids; ^1^with acid hydrolysis, ^2^ Calculated according to Kong and Adeola [31]

### Performance parameters

Animals were weighed pen-wise on d 1, 14, and 28 as well as individually on d 35 to determine BW. Average daily gain (ADG) and average daily feed intake (ADFI) were recorded at pen level at the end of each feeding phase. Feed conversion rate (FCR) was calculated from ADG and ADFI for each feeding phase and for the overall experimental period. Any clinical signs of illness or mortality were recorded daily, and dead birds were weighed to determine the losses.

### Sample collection

After 36 d of fattening, all broilers were slaughtered. Directly after slaughtering, digesta were collected from six representative broilers per pen (*n* = 216) being closest to the median of BW of the pen. Digesta from three gut sections [ileum (Meckel’s diverticulum until colon), caecum (whole caecum) and colon (whole colon)] were collected. Therefore, the intestinal tract was removed and opened. To gain enough digesta, homogenously mixed samples of six animals were pooled per pen, put into narrow mouth bottles and immediately frozen at − 20 °C until further analysis. Two representative broilers per pen (*n* = 72 animals), which had already been taken for digesta sampling, were selected to collect intestinal gut tissue samples. For purpose of histological analysis, samples from the jejunum were excised halfway between the duodenum and the Meckel’s diverticulum, and the ileum 3–6 cm above the ileocaecal junction. Samples were flushed thoroughly with ice-cold phosphate buffered saline to remove the entire digesta content, embedded in slotted cassettes and immersed in 4% paraformaldehyde (v/v) for 48 h. Excreta of all birds were collected pen-wise on d 34 for determination of AME_N_. For this purpose, excreta samples were frozen immediately at − 20 °C and calculation of AME_N_ was done according to Kong and Adeola [31].

### Chemical analyses of feed, excreta and digesta samples

Prior to analysis, ileal digesta samples were thawed at 4 °C and freeze-dried, whereas caecal and colonic digesta samples were analysed in fresh matter. *Hermetia illucens* larvae meal, diet and ileal digesta samples were ground to pass a 1-mm sieve and proximate composition was analysed according to the standard procedures of VDLUFA [32]. These analyses are: dry matter (DM; method no. 3.1.4), crude ash (CA; method no. 8.1.1), ether extract (EE; method no. 5.1.1), ether extract after acid hydrolysis (EEh; method no. 5.1.2) and crude fibre (CF; method no. 6.1.2). The gross energy (GE) content in feed and excreta was determined using an adiabatic bomb calorimetry (IKA C 200, IKA Werke GmbH & Co. KG, Staufen, Germany). To determine the nitrogen content, dumas combustion method (DuMaster 480, Büchi AG, Flawil, Switzerland) (method no. 4.1.2) [33] was used and multiplied by 6.25 to determine CP concentration. The amount of acid-detergent fibre (ADF) and ADF-linked protein was determined according to Licitra et al. [34]. Additionally, wet-ashing in a microwave oven (CEM Mars 6, CEM Corp. Matthews, NC, USA) was applied to analyse Ca and Na by flame atomic absorption spectrophotometry (AAnalyst200, Perkin Elmer Inc. Massachusetts, USA), and P photometrically (Tecan Group Ltd., Männedorf, Switzerland) using the vanado-molybdate method at 436 nm.

Finisher feed and ileal digesta samples were also analysed for titanium dioxide (TiO_2_) concentration as described by Leone et al. [35]. Therefore, 0.5 g of sample was mixed with a catalyst tablet and 25 mL concentrated sulphuric acid. The mixture was digested at 400 °C for 115 min on a block digesta. After removing and cooling the tubes, digestion was transferred to a volumetric flask and the volume was made up to 100 mL with distilled water. After filtration, 5 mL of each sample was blended with 1 mL of 1 mol/L sulphuric acid and 1 mL hydrogen peroxide (300 mL/L). Subsequently, measurement was made in comparison to a titanium sulphate standard at 405 nm using a UV-vis spectrophotometer (UV-1800, Shimadzu Corp., Kyoto, Japan). Total amino acid analyses of HI larvae meal and diets were performed by reverse-phase HPLC according to Waters AccQ. Tag method [36], after hydrolysis in 6 mol/L HCl for 24 h at 110 °C.

Determination of the fatty acid profile in feed was performed according to the 1-step methylation method of Sukhija and Palmquist [37]. Briefly, for extraction, 0.5 g of sample was treated with 3 mL of toluene including nonadecanoic acid as internal standard (concentration 1 mg/mL, Sigma-Aldrich, Munich, Germany). Samples were transmethylated by addition of 3 mL 5% methanol-hydrochloric acid solution to determine fatty acid as methyl esters (FAME). To avoid oxidation processes, samples were flushed with nitrogen for 30 s. Subsequently, tubes were closed gas-tight and heated in a water bath (at 70 °C) for 2 h while vortexing every 30 min. Following cooling for 30 min, a biphasic separation was obtained by adding 5 mL of 6% potassium carbonate solution and 1 mL toluene to each sample. Tubes were then shaken vigorously, vortexed and centrifuged at 175 × *g* for 5 min. The supernatant was then transferred to a 1.5-mL vial. The FAME were quantified by gas chromatography (Agilent Technologies 7890A, Waldbronn, Germany), equipped with a J&W HP-88 GC column (112-88A7, Agilent Technologies, Inc., Santa Clara, CA, USA; 100 m × 250 μm × 0.20 μm) and flame-ionization detector. Hydrogen was used as carrier gas at a constant pressure of 11 psi. The injector temperature was 250 °C and detector temperature 260 °C. The oven temperature was set to 100 °C for 5 min afterwards temperature was raised by 4 °C/min to 240 °C, which was then held for 30 min. Peaks were identified based on commercially available FAME mixtures (37-Component FAME Mix, Supelco Inc., Bellefonte, PA, USA). The results are expressed as the percentage (%) of total detected FAME.

$$ Fatty\kern0.28em acids\kern0.28em \left(\%\kern0.28em FAME\right)\kern0.28em =\frac{\left( area\kern0.28em peak\kern0.28em sample\right)}{\left( Sum\kern0.28em of\kern0.28em peak s\kern0.28em area\kern0.28em -\kern0.28em area\kern0.28em internal\kern0.28em s\tan dard\right)} $$

### Microbial metabolites

Analyses of the concentrations of biogenic amines in caecal and colonic digesta were performed according to Saarinen [38] using reverse-phase HPLC (Waters 2695e Separations Module, Waters, Milford, USA). For this purpose, a RP-18 column (InertClone™ 5 μm ODS (2) 150 Å, 250mm × 4.6 mm, Phenomenex, Aschaffenburg, Germany) was used and the detection was conducted by UV (Waters 2489 UV-visible detector, Waters, Milford, MA, USA). Data were calculated using the software Empower 3 (Waters, Milford, MA, USA). As eluents, 0.1 mol/L ammonia-acetate buffer (pH 5) (Carl Roth GmbH + Co. KG, Karlsruhe, Germany) and acetonitrile (HPLC grade; Carl Roth GmbH + Co. KG, Karlsruhe, Germany) were used. To determine the ammonia content, approximately 1.0 g of digesta sample was vortexed with 1.0 mL perchloric acid (1 mol/L). Subsequently, the mixture was allowed to settle for 10 min before 8 mL of double-distilled H_2_O was added and vortexed again. Samples were then shaken (POLYMAX 1040, Heidolph Instruments, Schwabach, Germany) for 1 h, before being centrifuged at room temperature for 10 min at 3215 × *g* (Centrifuge 5810R, Eppendorf, Wesseling-Berzdorf, Germany). Storage of the supernatant took place at − 20 °C. Before being analysed, thawed samples were centrifuged at 12,045 × *g* for 5 min (Minispin, Eppendorf, Wesseling-Berzdorf, Germany) at room temperature and supernatant was used for analysis of ammonia.

The ammonia concentration was analysed by preparing a mixture of 0.5 mL of salicylate-nitroprusside colour reagent (blend of equal parts of sodium hydroxide 0.3 mol/L, double-distilled H_2_O and salicylate-nitroprusside solution) and 0.25 mL of dichloroisocyanurate solution (0.050 g dichloroisocyanurate dissolved in 50 mL double-distilled H_2_O). To ensure a proper colouring reaction, 1.0 mL of diluted sample extract or standard solution (Ammonia standard solution ROTI®Star, Karlsruhe, Germany) was added immediately afterwards. Following incubation for 1.5 h at room temperature in the dark, the concentration of ammonia was analysed spectrophotometrically at 660 nm (Tecan Austria GmbH, Grödig, Austria).

The SCFA concentrations were determined by applying the method of Zhao et al. [39]. The digesta sample (~ 1 g) was weighed and 1 mL of 5 mmol/L internal standard solution (2-ethylbutyric-acid 99%, Sigma Aldrich, Steinheim, Germany) was added to reach a final concentration of 1 mmol/L. Subsequently, 4 mL of double-distilled water were added, the tubes were thoroughly mixed and put for 1 h on a shaker (Polymax 1040, Heidolph Instruments, Schwabach, Germany). Afterwards, HCl (5 mol/L) was used for a pH adjustment to 2–3. Subsequently, the tubes were centrifuged at 3215 × *g* for 20 min (Centrifuge 5810R, Eppendorf, Wesseling-Berzdorf, Germany) after 10 min of incubation. The supernatant was immediately stored at − 20 °C. For analysis, the samples were thawed and centrifuged at 12,045 × *g* for 20 min (Minispin, Eppendorf, Wesseling-Berzdorf, Germany). Acetic acid, propionic acid, isobutyric acid, butyric acid, isovaleric acid, valeric acid, isocapronic acid, heptanoic acid and hexanoic acid were separated and quantified using a gas chromatographic system (Agilent Technologies 7890A-G3440A-GC System, Agilent Technologies, Santa Clara, USA) with a C-18 capillary column (Agilent CP-Sil 5CB, length 25 m, diameter 0.32 mm) by a flame ionisation detection unit.

### Histomorphometry

For purpose of histological analysis, intestinal gut tissue samples were first dehydrated and embedded in paraffin wax blocks, before being sectioned at 5 μm thickness using a microtome (Leica RM2255, Leica Biosystems GmbH, Wetzlar, Germany) and mounted onto glass slides (Menzel-Gläser Superfrost-Plus, Thermo Scientific, Braunschweig, Germany). Sections were subsequently stained (Leica Auto-Stainer XL ST5010, Leica Biosystems GmbH, Nussloch, Germany) following the standard protocol for Alcian blue-periodic acid–Schiff. For visualisation, a light microscope (Leica DM 6000 B, Leica) and the software Leica Application Suite (Version 4.13, Leica) were used. Morphometric indices examined were: villus height (Vh; measured from the tip of the villus to the villus-crypt axis), villus width (Vw; at the crypt-villus axis), villus area (Va; as cross-sectional area of a villus measured above the villus-crypt axis) and crypt depth (Cd; from the base of the villus to the submucosa). Using measurement of Vh and Cd, their ratio (Vh:Cd) was calculated. Goblet cells were counted and are expressed as number of cells per 200 μm of villus epithelium. In addition, the thickness of the submucosa and *muscularis circularis* was determined. For all these indices, measurements were taken on six well oriented villi, their crypts, and the underlying tissue layer that were regularly distributed along the entire intestinal surface. The respective means of these six measurements were subsequently used for statistical analysis.

### Calculations

The following equation was used to calculate the apparent ileal digestibility (AID):

$$ \boldsymbol{AID}\kern0.5em \%=\left\{\mathbf{1}\hbox{-} \left[\left(\frac{\boldsymbol{T}\ {\boldsymbol{iO}}_{\boldsymbol{2}}\kern0.5em \%\kern0.5em \boldsymbol{diet}}{\boldsymbol{T}\ {\boldsymbol{iO}}_{\boldsymbol{2}}\kern0.5em \%\kern0.5em \boldsymbol{digesta}}\right)\kern0.5em \times \kern0.5em \right.\left(\frac{\boldsymbol{Nutrient}\kern0.5em \%\kern0.5em \boldsymbol{digesta}}{\boldsymbol{Nutrient}\kern0.5em \%\kern0.5em \boldsymbol{diet}}\right)\right\}\kern0.5em \times \kern0.5em \mathbf{100} $$

### Statistical analyses

The following statistical model was used:

$$ {Y}_{ij k}=\mu +{\alpha}_i+{\beta}_j+{\left(\alpha \times \beta \right)}_{ij}+{b}_k+{e}_{ij k}. $$

where *Y*_*ijk*_ is the dependent variable, μ the overall mean, α_i_ the fixed effect of protein source (*i* = SBM or HI larvae meal (15%)), *β*_*j*_ the fixed effect of fat substitution (*j* = soybean oil, 50/50 soybean oil and HI larvae fat or 100% HI larvae fat), *b*_*k*_ the random effect of pen (*k* = 1, 2, 3,..., 6) and *e*_*ijk*_ the residual error term. Statistics of performance parameters, digestibility coefficients, histological measurements and microbial metabolites were subjected to mixed model analysis using the PROC MIXED procedure of SAS v. 9.4 (Cary, NC, USA). Priorly, data were analysed for normal distribution using PROC UNIVARIATE. Significance was defined at *P* < 0.05, a trend at 0.05 ≤ *P* < 0.1 and results are expressed as least square means. Differences between the least squares means were post-hoc tested using an adjusted Tukey test.

## Results

### Animal performance

Performance data are presented in Table 4. Animals developed well during the entire experimental period and mortality was 5.0%, without differences between treatment groups.

**Table 4:** Summarised performance results of the starter, grower, and finisher period as well as the overall broiler trial (*n* = 6)

Items	Protein × Fat						SE	*P*-value	Protein (LSMean)		SE	*P*-value	Fat (LSMean)			SE	***P***-value
	S + 0 L	S + 50 L	S + 100 L	L + 0 L	L + 50 L	L + 100 L			S	L			0 L	50 L	100 L		
Starter period, d 1–14																	
Body weight, g	559^Z^	552^YZ^	539^BY^	564	553	564^A^	5.6	0.028	550	561	4.2	0.008	562^u^	553^uv^	551^v^	4.569	0.076
ADFI, g/bird	40	40	39	40	39	40	0.47	0.004^1^	39	39	0.35	0.693	40	40	39	0.388	0.379
ADG, g	37	36	35^B^	37	36	37^A^	0.41	0.030	36	37	0.30	0.009	37	36	36	0.332	0.091^1^
FCR, g/g	1.079^Y^	1.106^AZ^	1.094^YZ^	1.077	1.067^B^	1.076	0.007	0.011	1.093	1.074	0.005	< 0.001	1.078	1.087	1.085	0.006	0.298
Grower period, d 15–28																	
Body weight, g	2047	2059	1987	2047	2024	2041	24.6	0.188	2031	2037	15.28	0.756	2047	2041	2014	18.05	0.345
ADFI, g/bird	133	133	130	135	134	136	1.46	0.146	132	135	0.85	0.041	134	134	133	1.044	0.759
ADG, g	106	108	103	106	105	105	1.6	0.361	106	105	0.93	0.794	106	106	104	1.126	0.442
FCR, g/g	1.254	1.243	1.256	1.274	1.272	1.289	0.01	0.884	1.251	1.278	0.007	0.015	1.264	1.257	1.273	0.009	0.503
Finisher period, d 29–35																	
Body weight, g	2975	2908	2888	3039	2934	2982	53.8	0.744	2924	2985	39.81	0.103	3007	2921	2935	43.73	0.136
ADFI, g/bird	175	178	174	183	184	183	3.88	0.774	175	183	3.32	< 0.001	179	181	179	3.470	0.649
ADG, g	133	121	129	141	130	134	7.6	0.938	128	135	6.32	0.072	137	126	132	6.662	0.103
FCR, g/g	1.319	1.503	1.364	1.293	1.492	1.377	0.09	0.968	1.396	1.387	0.06	0.894	1.306^v^	1.498^u^	1.371^uv^	0.071	0.052
Overall period, d 1–35																	
ADG, g	84	82	81	86	83	84	1.5	0.741	82	84	1.13	0.102	85	82	83	1.246	0.137
ADFI, g	104	105	102	106	107	107	1.23	0.168	104	107	0.841	0.005	105	106	105	0.954	0.460
FCR, g/g	1.244	1.289	1.256	1.242	1.296	1.278	0.02	0.815	1.263	1.272	0.01	0.572	1.243^v^	1.293^u^	1.267^uv^	0.016	0.052

S, soybean meal; L, 15% of SBM crude protein substituted with HI larvae meal CP; S + 0 L, SBM + 0 HI larvae fat; S + 50 L, SBM + 50% HI larvae fat; S + 100 L, SBM+ 100% HI larvae fat; L + 0 L, HI larvae meal+ 0 HI larvae fat; L + 50 L, HI larvae meal+ 50% HI larvae fat; L + 100 L, HI larvae meal+ 100% HI larvae fat, *ADFI* Average daily feed intake, *ADG* Average daily gain, *FCR* Feed conversion ratio

^A,B^ Protein sources at the same HI larvae fat inclusion level differ if having different superscripts (*P* < 0.05)

^Y,Z^ HI larvae fat level within soybean feeding not sharing a common superscript differ (*P* < 0.05)

^u,v^ indicate differences by trend (0.05 ≤ *P* ≤ 0.10) for the main effect of fat source

^1^ post hoc test Tukey *P* > 0.10

*SE* Standard error

In the starter period, an interaction for the protein and fat source was observed for BW (*P* = 0.03), ADG (*P* = 0.03) and FCR (*P* = 0.01). Body weight and ADG increased in L + 100 L compared to S + 100 L. Regarding FCR, S + 50 L was higher than L + 50 L. Concerning the main effect protein, BW and ADG were affected with on average 2% higher BW for L groups compared to S groups. Similarly, FCR improved in L groups compared to S groups (*P* < 0.001). For fat source, a trend was detected for BW (*P* = 0.08) with 0 L being higher than 100 L. Average daily feed intake was not affected by interaction, protein or fat source in the starter period.

In the grower period, no interaction effect on performance parameters was observed. Referring to protein source, an effect for ADFI (*P* = 0.04) and FCR (*P* = 0.01) was detected. Feeding HI larvae meal as protein source increased ADFI compared to SBM, but impaired FCR. No effects of fat source on performance were detected in the grower period.

In the finisher period, performance was not affected by the interaction of the experimental factors protein and fat. Concerning the protein source, performance in terms of ADFI (*P* = 0.0005) and ADG (*P* = 0.07) increased or tended to increase in L groups compared to S groups. With regard to the effect of fat source, there was a trend for improved FCR in 0 L groups compared to 50 L groups (*P* = 0.05). Body weight was not affected in the finisher period.

In the overall period, animal performance parameter did not show interaction of protein and fat source. For the effect of protein source, ADFI was higher in L groups compared to S groups (*P* = 0.005). Fat source tendentially impaired FCR in the overall period in 50 L groups compared to 0 L groups (*P* = 0.05).

### Apparent ileal digestibility

Table 5 illustrates the AID of crude nutrients. A trend for an interaction of protein and fat source was observed concerning the AID of fat, being improved in L + 50 L compared to S + 50 L. Protein source affected the AID of DM (*P* < 0.01) OM (*P* < 0.01) and fat (*P* = 0.02), all being increased by HI larvae feeding. Fat source tended (*P* = 0.08) to affect the AID of CP, showing improved CP digestibility in 100 L compared to 0 L.

**Table 5:** Apparent ileal digestibility of broilers fed with HI larvae meal and fat

Items, %	Protein × Fat						SE	***P***-value	Protein (LSMean)		SE	***P***-value	Fat (LSMean)			SE	***P***-value
	S + 0 L	S + 50 L	S + 100 L	L + 0 L	L + 50 L	L + 100 L			S	L			0 L	50 L	100 L		
Dry matter	63.5	63.9	63.6	66.4	67.8	66.0	1.63	0.611	63.6	66.7	0.57	< 0.001	64.9	65.8	64.8	0.692	0.347
Organic matter	67.9	68.3	68.1	70.3	71.8	70.1	1.01	0.543	68.1	70.7	0.63	< 0.001	69.1	70.0	69.1	0.721	0.285
Crude protein	73.1	73.2	73.8	71.4	72.4	74.8	1.18	0.350	73.4	72.8	0.67	0.488	72.3^v^	72.8^uv^	74.3^u^	0.799	0.081
Fat	88.7	85.7^y^	88.7	88.7	92.1^z^	91.0	2.26	0.084	87.7	90.6	1.48	0.022	88.7	88.9	89.9	1.649	0.712

S, soybean meal; L; 15% of SBM crude protein substituted with HI larvae meal CP; S + 0 L, SBM + 0HI larvae fat; S + 50 L, SBM + 50% HI larvae fat; S + 100 L, SBM+ 100% HI larvae fat; L + 0 L, HI larvae meal+ 0HI larvae fat; L + 50 L, HI larvae meal+ 50% HI larvae fat; L + 100 L, HI larvae meal+ 100% HI larvae fat

^y,z^ Protein sources at the same HI larvae fat (50, 100%) level differ by trend (0.05 ≤ *p* ≤ 0.10) if having different superscripts

^u,v^ indicate differences by trend (0.05 ≤ *P* ≤ 0.10) for the main effect of fat source

*SE* Standard error

### Microbial metabolites and intestinal morphometric indices

Concentrations of biogenic amines and ammonia in the caecum are given in Table 6. No interaction effect on microbial metabolites determined in the caecum was observed. Concerning the effect of protein source, agmatine (*P* < 0.01), ethanolamine (*P* = 0.02), spermidine (*P* < 0.01), spermine (*P* < 0.01) and ammonia (*P* < 0.01) concentrations were affected. This effect manifested itself with higher concentrations in S groups compared to L groups for agmatine, spermidine, spermine and ammonia. In contrast, the ethanolamine concentration was higher for L groups compared to S groups. Moreover, protein source tended to affect methylamine and putrescine concentrations (*P* = 0.06 each) being lower with HI larvae meal inclusion. Regarding the main effect of fat, a trend was determined for the concentration of spermine being higher in 0 L groups compared to 100 L groups (*P* = 0.07). No effect was observed for cadaverine, histamine and tyramine.

**Table 6:** Biogenic amines and ammonia in caecal digesta of broilers

Items	Protein × Fat						SE	***P***-value	Protein (LSMean)		SE	***P***-value	Fat (LSMean)			SE	***P***-value
	S + 0 L	S + 50 L	S + 100 L	L + 0 L	L + 50 L	L + 100 L			S	L			0 L	50 L	100 L		
Biogenic amines, mg/kg fresh matter																	
Agmatine	244	293	274	222	214	213	26.18	0.237	270	216	22.51	< 0.001	233	254	243	23.48	0.460
Ethanolamine	6.00	9.93	4.51	10.1	9.50	10.7	1.715	0.121	6.81	10.1	1.114	0.017	8.04	9.71	7.61	1.287	0.387
Methylamine	7.51	7.45	7.06	6.51	6.43	5.41	0.821	0.895	7.34	6.12	0.522	0.065	7.01	6.94	6.24	0.611	0.550
Putrescine	1.40	1.30	1.68	1.13	0.31	0.73	0.463	0.670	1.46	0.72	0.282	0.056	1.27	0.80	1.20	0.336	0.539
Cadaverine	4.17	5.69	3.82	4.86	3.57	3.70	0.796	0.107	4.56	4.04	0.590	0.343	4.52	4.63	3.76	0.648	0.365
Histamine	1.16	1.19	1.05	1.14	1.06	1.07	0.063	0.476	1.14	1.09	0.038	0.357	1.14	1.28	1.06	0.046	0.345
Tyramine	1.12	1.09	3.11	1.10	1.83	2.21	1.321	0.825	1.77	1.71	0.763	0.958	1.11	1.46	2.66	0.934	0.479
Spermidine	220	238	234	185	187	179	15.11	0.674	231	183	11.83	< 0.001	202	212	206	12.73	0.689
Spermine	13.7	11.6	11.0	9.53	10.5	8.53	0.777	0.166	12.1	9.51	0.448	< 0.001	11.6^u^	11.0^uv^	9.75^v^	0.549	0.067
Ammonia, mmol/kg fresh matter	23.2	24.0	22.6	20.2	21.3	19.1	1.140	0.916	23.3	20.2	0.853	< 0.001	21.7	22.6	20.9	0.933	0.181

S, soybean meal; L; 15% of SBM crude protein substituted with HI larvae meal CP; S + 0 L, SBM + 0 HI larvae fat; S + 50 L, SBM + 50% HI larvae fat; S + 100 L, SBM+ 100% HI larvae fat; L + 0 L, HI larvae meal+ 0 HI larvae fat; L + 50 L, HI larvae meal+ 50% HI larvae fat; L + 100 L, HI larvae meal+ 100% HI larvae fat

^u,v^ indicate differences by trend (0.05 ≤ *P* ≤ 0.10) for the main effect of fat source

*SE* Standard error

Table 7 shows the concentration of SCFA in caecal digesta. Isocaproic acid, hexanoic acid and heptanoic acid concentrations were additionally analysed but values were below detection limit. No interaction effect on the determined SCFA concentrations was observed. With regard to the protein source, an effect on the concentration of butyric acid was monitored with higher concentrations in S groups compared to L groups (*P* = 0.03). The fat source tendentially affected the concentration of acetic acid increasing in 100 L groups compared to 50 L groups (*P* = 0.07). The concentration of propionic, valeric, isobutyric and isovaleric acid were neither affected by an interaction nor by the main effects protein and fat source.

**Table 7:** Short-chain fatty acid (SCFA) concentrations in caecal digesta of broilers

Items	Protein × Fat						SE	***P***-value	Protein (LSMean)		SE	***P***-value	Fat (LSMean)			SE	***P***-value
	S + 0 L	S + 50 L	S + 100 L	L + 0 L	L + 50 L	L + 100 L			S	L			0 L	50 L	100 L		
SCFA, mmol/kg fresh matter																	
Acetic acid	102	99.1	120	113	97.0	101	8.168	0.035^1^	107	104	6.888	0.425	107^uv^	98.0^v^	110^u^	7.229	0.070
Propionic acid	14.0	12.3	13.4	15.1	13.7	12.5	2.036	0.695	13.2	13.7	1.633	0.682	14.6	13.0	12.9	1.742	0.483
Butyric acid	17.5	19.9	23.1	18.3	16.4	17.3	1.667	0.113	20.2	17.3	1.119	0.031	17.9	18.2	20.2	1.278	0.262
Valeric acid	2.02	2.22	2.12	1.91	2.30	1.82	0.314	0.756	2.02	2.01	0.239	0.579	1.97	2.26	1.97	0.260	0.406
BCFA, mmol/kg fresh matter																	
Isobutyric acid	1.82	1.76	2.07	1.64	2.03	1.37	0.596	0.429	1.88	1.68	0.516	0.501	1.73	1.90	1.72	0.537	0.862
Isovaleric acid	0.29	0.31	0.30	0.16	0.32	0.15	0.181	0.657	0.30	0.21	0.165	0.274	0.22	0.32	0.22	0.169	0.527

S, soybean meal; L; 15% of SBM crude protein substituted with HI larvae meal CP; S + 0 L, SBM + 0 HI larvae fat; S + 50 L, SBM + 50% HI larvae fat; S + 100 L, SBM+ 100% HI larvae fat; L + 0 L, HI larvae meal+ 0% HI larvae fat; L + 50 L, HI larvae meal+ 50% HI larvae fat; L + 100 L, HI larvae meal + 100% HI larvae fat

^u,v^ indicate differences by trend (0.05 ≤ *P* ≤ 0.10) for the main effect of fat source

^1^post hoc test Tukey *P* > 0.10

*SE* Standard error

The concentrations of biogenic amines in the colonic digesta are presented in Table 8. Neither an interaction effect nor an effect of the protein source was observed. Concerning the fat source, HI larvae fat increased the concentration of agmatine (*P* = 0.03) when feeding 50% HI larvae fat compared to 0 HI larvae fat.

**Table 8:** Biogenic amines in colonic digesta of broilers

Items	Protein × Fat						SE	***P***-value	Protein (LSMean)		SE	***P***-value	Fat			SE	***P***-value
	S + 0 L	S + 50 L	S + 100 L	L + 0 L	L + 50 L	L + 100 L			S	L			0 L	50 L	100 L		
Biogenic amines, mg/kg fresh matter																	
Agmatine	463	518	397	347	866	531	116.3	0.106	460	582	79.07	0.165	405^V^	692^U^	464^UV^	89.85	0.027
Ethanolamine	21.9	22.9	26.2	22.4	40.7	29.1	5.919	0.300	23.7	30.7	3.458	0.155	22.2	31.8	27.7	4.210	0.281
Methylamine	3.52	4.63	4.63	4.86	3.63	4.37	1.420	0.636	4.26	4.29	0.991	0.978	4.19	4.13	4.50	1.114	0.949
Putrescine	2.69	2.45	3.48	1.05	2.51	4.13	1.796	0.770	2.88	2.56	1.194	0.817	1.87	2.48	3.81	1.370	0.495
Cadaverine	8.58	12.3	15.3	15.1	9.77	8.14	3.462	0.076^1^	12.1	11.0	2.515	0.663	11.9	11.1	11.7	2.782	0.959
Spermidine	59.5	57.9	61.8	67.3	58.1	82.9	11.76	0.616	59.8	69.4	7.90	0.278	63.4	58.0	72.3	9.021	0.410
Spermine	56.6	56.1	53.8	48.4	58.8	49.4	4.698	0.473	55.5	52.2	3.00	0.369	52.5	57.4	51.6	3.50	0.382

S, soybean meal; L; 15% of SBM crude protein substituted with HI larvae meal CP; S + 0 L, SBM + 0 HI larvae fat; S + 50 L, SBM + 50% HI larvae fat; S + 100 L, SBM+ 100% HI larvae fat; L + 0 L, HI larvae meal+ 0 HI larvae fat; L + 50 L, HI larvae meal+ 50% HI larvae fat; L + 100 L, HI larvae meal+ 100% HI larvae fat

^U,V^ indicate differences (*P* < 0.10) for the main effect of fat source

^1^post hoc test Tukey *P* > 0.10

*SE* Standard error

The analysis of the intestinal morphometric indices in the jejunum (Table 9) showed an interaction of protein and fat for Va (*P* = 0.01), which increased in L +  100 L compared to S + 100 L, as well as increased in 50 L compared to 0 L groups when fed HI larvae meal, but not when fed SBM. Regarding the protein source, Va (*P* = 0.02) and Vw (*P* = 0.03) were affected with L groups being higher than S groups. Fat source affected Va (*P* = 0.03) and tended to affect Vh (*P* = 0.06) and the ratio of Vh:Cd (*P* = 0.09). Villus area and Vh were higher in 50 L groups compared to 0 L groups. With respect to the ratio of Vh:Cd, 50 L tended to have a better ratio than 100 L. The variables crypth depth, number of goblet cells, mucosa, submucosa and *tunica muscularis* were neither affected by an interaction, nor protein or fat in the jejunum. In the ileum, an interaction was observed for Vw (*P* = 0.03), which was higher in L + 0 L compared to L + 100 L. No effect of protein source was observed. Concerning the fat source, a trend was observed for greater thickness of the submucosa in 50 L compared to 100 L groups. No interaction or main effects were observed for Vh, Va, Vh:Cd, number of goblet cells, mucosa and *tunica muscularis* in the ileum.

**Table 9:** Intestinal morphometric indices of broilers

Items	Protein × Fat						SE	***P***-value	Protein (LSMean)		SE	***P***-value	Fat			SE	***P***-value
	S + 0 L	S + 50 L	S + 100 L	L + 0 L	L + 50 L	L + 100 L			S	L			0 L	50 L	100 L		
Jejunum																	
Villus height, μm	1114	1179	1008	994	1214	1157	71.06	0.114	1100	1122	45.78	0.678	1054^v^	1197^u^	1082^uv^	52.95	0.065
Villus width, μm	188	193	172	197	222	204	14.43	0.641	184	207	9.149	0.033	192	207	188	10.65	0.288
Villus area, mm^2^	0.19	0.19	0.15^B^	0.16^Y^	0.24^Z^	0.22^A,YZ^	0.015	0.015	0.18	0.21	0.001	0.018	0.17	0.21	0.18	0.011	0.035
Crypt depth, μm	135	125	137	120	133	137	7.952	0.314	132	130	4.795	0.701	127	129	137	5.713	0.388
Villus height/crypt depth	8.57	9.85	7.68	8.53	9.56	8.54	0.808	0.721	8.70	8.88	0.495	0.769	8.55^uv^	9.70^u^	8.11^v^	0.585	0.093
Goblet cells villus, n/200 μm	19.1	15.8	15.5	16.0	15.6	15.3	1.319	0.393	16.8	15.6	0.808	0.238	17.5	15.7	15.4	0.956	0.184
Mucosa, μm	1356	1395	1243	1220	1471	1345	84.68	0.292	1331	1345	47.52	0.832	1288	1433	1294	58.62	0.139
Submucosa, μm	33.9	33.0	34.8	29.8	34.4	34.7	1.956	0.302	33.9	33.0	1.929	0.537	32	34	35	1.414	0.294
Tunica muscularis circular layer, μm	136	136	138	137	134	134	9.637	0.971	137	135	6.150	0.787	137	135	136	7.142	0.984
Ileum																	
Villus height, μm	523	556	492	566	513	564	33.64	0.189	524	548	20.91	0.369	545	535	528	24.72	0.877
Villus width, μm	155	153	165	174^y^	157	140^z^	8.434	0.034	157	157	4.869	0.947	164	155	152	5.964	0.351
Villus area, mm^2^	0.07	0.08	0.07	0.09	0.07	0.08	0.006	0.379	0.07	0.08	0.003	0.363	0.08	0.07	0.08	0.004	0.541
Crypt depth, μm	119	143	134	144	134	143	7.144	0.072^1^	132	140	4.125	0.159	132	138	138	5.051	0.562
Villus height/crypt depth	4.56	3.89	3.72	3.97	3.90	3.97	0.245	0.184	4.05	3.94	0.156	0.567	4.26	3.89	3.84	0.183	0.148
Goblet cells villus, n/200 μm	30	28	26	27	30	28	3.511	0.442	28	28	2.893	0.760	28	29	27	3.059	0.675
Mucosa, μm	676	725	629	722	671	704	39.17	0.215	677	699	23.64	0.475	699	698	667	28.33	0.635
Submucosa, μm	44.7	48.3	38.8	41.5	47.6	43.4	3.115	0.423	44.0	44.2	1.902	0.923	43.1^uv^	47.9^u^	41.1^v^	2.267	0.072
Tunica muscularis circular layer, μm	277	270	239	227	278	264	24.08	0.238	262	256	14.97	0.770	252	274	252	17.70	0.541

S, soybean meal; L; 15% of SBM crude protein substituted with HI larvae meal CP; S + 0 L, SBM + 0 HI larvae fat; S + 50 L, SBM + 50% HI larvae fat; S + 100 L, SBM+ 100% HI larvae fat; L + 0 L, HI larvae meal+ 0 HI larvae fat; L + 50 L, HI larvae meal+ 50% HI larvae fat; L + 100 L, HI larvae meal+ 100% HI larvae fat

^A,B^ Protein sources at the same HI larvae fat inclusion level differ if having different superscripts (*P* < 0.05)

^Y,Z^ HI larvae fat level within protein sources not sharing a common superscript differ (*P* < 0.05)

^y,z^ HI larvae fat level within HI larvae feeding not sharing a common superscript differ by trend (0.05 ≤ *P* ≤ 0.10)

^u,v^ indicate differences by trend (0.05 ≤ *P* ≤ 0.10) for the main effect of fat source

^1^post hoc test Tukey *P* > 0.10

*SE* Standard error

## Discussion

### Animal performance

Our study showed that the use of HI larvae meal and fat did not negatively affect the performance of the animals. Remarkably, in the particularly important starter phase, the use of HI larvae meal even had a positive effect on chicken growth as evidenced by increased BW and ADG. For HI larvae fat, this was also true when exclusively fed together with HI larvae meal, which altogether confirms our hypothesis that HI larvae meal and fat can be fed without detrimental effects on performance. Regarding the effect of HI larvae meal as protein source, this coincides with the results of other studies showing that used in small amounts (5–10% of fresh matter in the diet), HI larvae meal leads to similar or even improved animal performance [22, 23, 25, 40].

The substitution of soybean oil with up to 100% of HI larvae fat has previously been shown to allow similar broiler performance [26, 28]. It has been stated that fatty acid digestibility increases with the degree of unsaturation and Tancharoenrat et al. [41] found a lower digestibility of palmitic and stearic acid compared to oleic and linoleic acids. Regarding oleic acid, there was a slightly higher concentration, i.e. one percentage unit, in L + 100 L compared to S + 100 L diet, which might have contributed to better performance in the starter phase. Furthermore, synergistic effects of blending vegetable and animal fats, as a result of an improved ratio of unsaturated to saturated fatty acids [42], cannot serve as an explanation for the improved performance observed in the present experiment, since first this ratio did not differ between these treatment groups in the starter phase (Table 3). Second, differences did occur when feeding HI larvae fat solely and were better for the combination of HI larvae fat with HI larvae meal. An explanation for the improved performance in the starter phase could be related to the limited capacity for fat digestion in young chickens. It is known that bile secretion and recirculation are low in the early life of broilers [18]. Since MCFA digestion does not depend on the formation of bile acid-mediated micelles [12], feeding HI larvae fat, which is rich in MCFA, may indeed be advantageous in broiler production. Likewise, the slightly higher lauric acid concentration in L + 100 L compared to S + 100 L would support the explanation of this mode of action also for the HI larvae meal, as evidenced by the higher fat digestibility when this protein source was fed.

Furthermore, it was noticeable that ADFI increased in L groups compared to S groups during grower, finisher, and the overall experimental period. This observed increased ADFI could be due to birds’ originally entomophagous food habit. This matches observations of Cullere et al. [43], who observed that quails tended to consume more feed if HI larvae meal was included. A similar behaviour was also observed by Nascimento et al. [44], determining a preference of broilers towards *Tenebrio molitor* meal in a cafeteria-type feeding. However, the opposite has previously been observed by Kieronczyk et al. [27], where broilers consumed less feed with higher HI larvae fat inclusion, which nevertheless resulted in a better FCR. However, as far as the present study is concerned, the higher ADFI did not lead to a higher ADG, which is why the FCR got worse in the grower period. Since there are yet no studies available investigating the effects of simultaneously feeding separated HI larvae meal and fat to broilers, direct comparisons of the observed interaction with other data are not yet possible.

### Apparent ileal digestibility

Although the higher digestibility of DM, OM and ether extracts in the HI larvae meal diets has to be considered positive, it did only lead to a numerically higher performance in the finisher phase, when digestibility was measured, which is important from an economic point of view. When considering the results, however, it must be borne in mind that the present diets in general fulfilled or even exceeded the nutrient requirements, such as CP, lysine and AME_N_ concentrations. An improved digestibility likely could have exerted an effect on performance if the nutrient supply was limited or in shortage of the requirements. Provided the improved digestibility in the finisher phase can be extrapolated to all feeding phases, this may explain the enhanced performance of birds in the starter phase, in which high nutrient digestibility is especially important due to an immature gut development [18].

In terms of AID of CP, the present results fall neatly into those of previous studies suggesting an equal ileal CP digestibility when HI larvae meal accounted for low amounts (5%) in broiler diets [25]. Feeding broiler quails, Cullere et al. [43] found no effects of HI larvae meal (10–15% in the diet) on AID of nutrients except for a lower ether extract digestibility.

The lack of fat source-related effects on AID was rather surprising, as the different FA composition of HI larvae oil compared to soybean oil would have suggested changes in fat digestibility. On the one hand, HI larvae fat is known to contain MCFA and so the lauric acid content in the present finisher diets increased almost thirtyfold and twelvefold from 0 to 100% HI larvae fat (Table 3). As already stated, MCFA do not require bile acid-mediated micelle formation to be soluble in the aqueous phase [12], are passively absorbed and thus an immediate energy source for enterocytes and are therefore regarded readily available for the animal [12, 16]. On the other hand, this advantageous effect of MCFA and lauric acid may have been counteracted by the high share of longer saturated FA, which more than doubled in the finisher feeds from 0 to 100% HI larvae fat. Previous reports suggested poorer absorption of animal fats due to their highly bile-acid dependent digestion [18].

Additionally, synergistic effects have been reported for the blend of vegetable oils and animal fats improving fat digestion [18]. In the present study, the ratio of unsaturated to saturated FA in the diet has at least halved over all feeding phases within protein sources from groups with 0 larvae fat to 100% larvae fat. Contrary to the aforementioned observations of previous studies [12, 16, 18], we have neither seen an increase in digestibility due to lauric acid or a decrease due to saturated FA in 100% HI larvae fat groups, nor an improvement in the 50 L groups. In future research projects, however, it is worth investigating digestibility in the starter phase as well, to assess potential differences in this crucial phase of broiler production.

### Microbial metabolites in the gut (biogenic amines, ammonia and SCFA)

Nutrients escaping the host digestion and absorption in the small intestine are subsequently exposed to microbial fermentation by the caecal microbiota [45], whose fermentation profile can provide valuable information about the metabolic response of the intestinal microorganisms to nutritional influences [46]. The key findings concerning microbial metabolites, namely higher concentrations of agmatine, spermidine, spermine, ammonia and a trend for higher putrescine concentrations in S groups, were surprising, since present data on CP AID did not differ between treatments, which means that no increased flow of undigested protein was expected. Consequently, other factors must have been caused the changes observed in microbial fermentation.

Merging the observations concerning biogenic amines with the findings related to SCFA analyses, i.e. an interaction of protein and fat on acetate and higher butyrate concentrations in SBM groups, some explanatory approaches arise. The first possible explanation for decreased putrefaction products in digesta of HI larvae fed groups may be chitin, which is found in the exoskeleton of insects and may act as (potential) prebiotic [47]. There is evidence that the addition of shrimp meal as source of chitin is able to beneficially shape caecal fermentation from protein to carbohydrate fermentation, thereby decreasing the caecal ammonia concentration in broilers [48]. This explanation also dovetails nicely with the studies of Biasato et al. [49] and Borrelli et al. [47], who concluded that an increase in SCFA-producing bacteria during HI larvae meal feeding may be related to the bacteria’s ability to degrade chitin. Moreover, similar observations, meaning a shift from protein to carbohydrate fermentation, accompanied by a reduced formation of biogenic amines and ammonia with increasing fermentable carbohydrates, have been made previously in pigs [50].

Secondly, the simultaneous observation of a tendency for higher putrescine and ammonia concentrations in SBM groups along with increased butyrate concentrations when feeding solely SBM may indicate a causal relation. Since those measured metabolite concentrations are not only the result of production but also absorption [51, 52], it thus remains questionable whether the higher caecal butyrate concentration derives from a higher production, or if it is rather the result of an accumulation due to impaired butyrate absorption. Interestingly, putrescine and ammonia have been shown to downregulate the expression of specific butyrate transporters (monocarboxylate transporter) in pigs, resulting in reduced energy supply to colonocytes [53, 54]. Thus, increased levels of putrescine and ammonia in SBM groups may have resulted in a downregulation of butyrate transporters, which may explain the higher amount of butyrate in SBM compared to HI larvae meal groups. Thus, on the one hand, an increased butyrate concentration can be regarded as positive in terms of improved energy provision for enterocytes [55]. On the other hand, it seems conceivable that increased butyrate concentrations in SBM groups result from reduced absorption, due to the above-mentioned downregulation of the monocarboxylate transporter, which would diminish its metabolic meaning.

Contrary to our hypothesis, we have not seen effects of the fat source on the concentrations of microbial metabolites, apart from a trend for less spermine concentrations in the caecum. This was unexpected, since previous studies indicated an antimicrobial activity of lauric acid [9]. However, there are contradictory results on the effects of MCFA in the distal gastrointestinal tract. On the one hand, it is assumed that MCFA are largely absorbed in the proximal intestine [12], which may reduce their presence in the hindgut. On the other hand, Wu et al. [21] showed that both the composition of the caecal microbiome and its fermentation profile changed through supplemented lauric acid to the diet.

Although the level of potentially harmful microbial fermentation products decreased with HI larvae meal inclusion, it remains to be elucidated why the butyrate level increased in the SBM group, which makes a more in-depth investigation of the microbiome and the metabolic mechanisms related to this effect necessary.

### Histology

With its primary function to digest and subsequently absorb nutrients, the small intestine has a fundamental role for improving animal performance. In the present study, intestinal morphometric indices were analysed in the jejunum and ileum, since both segments are essential for fat digestion, i.e. the jejunum constitutes the major site for fat absorption [18], although changes in fat utilisation have mainly been associated with an increased absorption in the ileum [56]. The present analysis revealed that the jejunal Va was higher in L + 100 L compared to S + 100 L. Therefore, this was like the pattern observed for performance characteristics in the starter period, when both ADG and BW were higher in L + 100 L than S + 100 L. Assuming that the effect on Va was already present during starter phase, it may explain the improved animal performance in L + 100 L, since these animals would have a higher absorptive surface in the jejunum.

Apart from a slight tendency for the fat source to influence Vh:Cd in the jejunum, there was an absence of differences in Cd and Vh:Cd between treatments in both gut segments. This observation can be considered positive, as these indices are used to evaluate the turnover rate of the intestinal mucosa [57]. Thereby, deeper crypts indicate an increased cell turnover, since the stem-cell zone in the crypts is the starting point for cell renewal [58]. As an increased turnover rate means higher requirements for energy, which is then not available for muscle growth, the findings on Cd and Vh:Cd suggest that feeding the present amounts of HI larvae meal and fat to broilers will not change energy provision for growth. However, it has been shown by others that higher amounts of HI larvae meal negatively affect intestinal morphology in terms of shorter villi, deeper crypts and lower Vh:Cd [22, 59].

Another important parameter for assessing intestinal integrity are the goblet cells or the mucus secreted by them. The mucus produced serves the epithelium as protective barrier against physical and chemical damage [60] and operates as lubrication and transport medium between the contents of the lumen and the epithelium [61]. The present evaluation showed that the feeding of HI larvae meal and fat had no effect on the number of goblet cells in the jejunum and ileum. Based on the assumption that this number can indeed be used to infer the secretion activity of mucins, a similar level of mucus production can be deduced. Thus, no antinutritional factors seemed to be present in HI larvae protein and fat, which otherwise would have damaged the mucous epithelial protective barrier or which would have necessitated a higher level of mucus protection [60]. Based on an unchanged thickness of the *tunica muscularis* between the experimental groups, it can be assumed that intestinal peristalsis and thus contact of the luminal contents with the mucosa and ultimately nutrient absorption were undiminished [62]. Generally, the lack of substantial effects in the ileum is consistent with previous studies showing no alterations when feeding low amounts of HI larvae meal or up to 100% HI larvae fat to broiler chickens [25, 26].

## Conclusions

The present study was the first to test the simultaneous use of HI larvae meal and HI larvae fat, administered separately, in broiler feeding. The performance results suggest the possibility of replacing up to 15% CP from SBM with HI larvae meal and up to 100% soybean oil with HI larvae fat without impairments in animal performance. The improved animal performance in terms of increased ADG and BW, observed in the starter period, shows that the use of HI larvae meal and fat is not only an adequate substitute for SBM, but can even have positive effects. This may be attributed to a higher AID, as indicated by improved AID when feeding HI larvae meal as protein source in the finisher phase. In addition, the use of HI larvae protein has partially reduced the concentration of potentially harmful microbial metabolites, which may be regarded as positive in terms of gut health. To confirm the assumption that the improved performance observed in the starter period is attributed to a higher digestibility, it would be useful to explicitly examine the digestibility in the starter phase in the future. Furthermore, assessment of the gut microbiota may help to understand the observed changes in microbial metabolites and should therefore be conducted in future studies.

## Data Availability

The datasets used and analysed during the current study are available from the corresponding author on reasonable request.

## Abbreviations

AA: Amino acids
ADF: Acid-detergent fibre
ADFI: Average daily feed intake
ADG: Average daily gain
ADIN: Acid-detergent insoluble nitrogen
AID: Apparent ileal digestibility
AME_N_: Apparent metabolizable energy corrected for nitrogen
BW: Body weight
CP: Crude protein
FAME: Fatty acid methyl ester
FCR: Feed conversion rate
HI: *Hermetia illucens*
MCFA: Medium-chain fatty acids
SCFA: Short-chain fatty acids
SBM: Soybean meal
Va: Villus area
Vh: Villus height
Vh:Cd: Villus height:Crypt depth
Vw: Villus width
